# Protective effect of pioglitazone on morphine-induced neuroinflammation in the rat lumbar spinal cord

**DOI:** 10.1186/s12929-015-0187-2

**Published:** 2015-09-23

**Authors:** Mohammad Charkhpour, Hamed Ghavimi, Saeed Ghanbarzadeh, Bahman Yousefi, Arash Khorrami, Mehran Mesgari, Kambiz Hassanzadeh

**Affiliations:** Department of Pharmacology and Toxicology, Faculty of Pharmacy, Tabriz University of Medical Sciences, Tabriz, Iran; Department of Pharmacology and Toxicology, Faculty of Pharmacy, Zanjan University of Medical Sciences, Zanjan, Iran; Department of Pharmaceutics, Faculty of Pharmacy, Tabriz University of Medical Sciences, Tabriz, Iran; Department of Biochemistry and Clinical Laboratories, Faculty of Medicine, Tabriz University of Medical Sciences, Tabriz, Iran; Student Research Committee, Tabriz University of Medical Sciences, Tabriz, Iran; Drug Applied Research Center, Tabriz University of Medical Sciences, Tabriz, Iran; Cellular and Molecular Research Center and Department of Physiology and Pharmacology, Faculty of Medicine, Kurdistan University of Medical Sciences, Sanandaj, Iran

**Keywords:** Pioglitazone, Morphine, Neuroinflammation, GW-9662, PPAR-γ

## Abstract

**Background:**

Morphine-induced tolerance is associated with the spinal neuroinflammation. The aim of this study was to explore the effects of oral administration of the pioglitazone, the peroxisome proliferator activated receptor gamma (PPAR-γ) agonist, on the morphine-induced neuroinflammation in the lumbar region of the male Wistar rat spinal cord.

**Results:**

Co-administration of the pioglitazone with morphine not only attenuated morphine-induced tolerance, but also prevented the up-regulation of pro-inflammatory cytokines (tumor necrosis factor alpha, interleukin-1beta, and interleukin 6) and nuclear factor-kappa B activity. Administration of the GW-9662 antagonized the above mentioned effects of the pioglitazone.

**Conclusions:**

It is concluded that oral administration of the pioglitazone attenuates morphine-induced tolerance and the neuroinflammation in the lumbar region of the rat spinal cord. This action of the pioglitazone may be, at least in part, due to an interaction with the spinal pro-inflammatory cytokine expression and the nuclear factor-kappa B activity.

## Background

Our recent studies demonstrated that administration of the pioglitazone prevents the morphine antinociception tolerance and withdrawal symptoms in the rats [1, 2]. Morphine is one of the most widely used analgesics for relieving severe pain. Despite the known side-effects of the opioids, the clinical utility of opioid analgesics is often hampered by the development of analgesic tolerance that necessitates dose escalation. To date, several mechanisms have been proposed to contribute in the morphine analgesic tolerance such as: the N-methyl-D-aspartate (NMDA) receptors activation [3], down-regulation of the spinal glutamate transporters [4], the spinal glial activation, and the release of the pro-inflammatory cytokines [5] and apoptosis [6]. It is highly confirmed that morphine activates the glial cells during prolonged exposure [7, 8]. In this regard, it is recognized that chronic administration of the morphine augments the production of the pro-inflammatory cytokines including: tumor necrosis factor alpha (TNF-α), interleukin (IL)-1β and IL-6 in the central nervous system (CNS) [5]. The glial derived pro-inflammatory cytokines increase the neuronal excitability [8], sensitize the pain transmission neurons [9], up-regulated the NMAD receptors [10], down-regulated the glutamate transporters [4] and oppose opioid-induced analgesia [5].

Accumulating evidences have suggested that targeting of the spinal glial activation [11], hampering the pro-inflammatory cytokines expression [12] inhibition of the nuclear factor kappa B (NF-κB) activity [13] and antagonizing the pro-inflammatory cytokine receptors [5] may be considered as important strategies for preventing the morphine analgesic tolerance. In this regard, Shen et al. demonstrated that the intrathecal administration of the etanercept restored the antinociceptive effect of the morphine by inhibition the spinal pro-inflammatory cytokines expression [12]. Moreover, Tai et al. indicated that the amitriptyline attenuated the morphine analgesic tolerance by inhibition of the spinal pro-inflammatory cytokine expression [14].

The peroxisome proliferator activated receptor gammas (PPAR-γ) are the orphan receptors in the CNS and the immune system, which target several downstream genes, involved in inflammation [15]. The pioglitazone is one of the PPAR-γ agonists with a good profile of blood brain barrier penetration [16]. Pioglitazone exerts its anti-inflammatory effects through suppression of the glial cells functions [17, 18], and regulation of the NF-κB activity [19]. Recently, the pioglitazone has received increasing attention in addiction [20]. The clear evidence for a role of pioglitazone in the abused drug addiction came from of Stopponi et al.’s current study. The later study showed that pioglitazone inhibits the alcohol consumption and hampers the expression of the alcohol withdrawal signs [21].

**Fig. 1:**
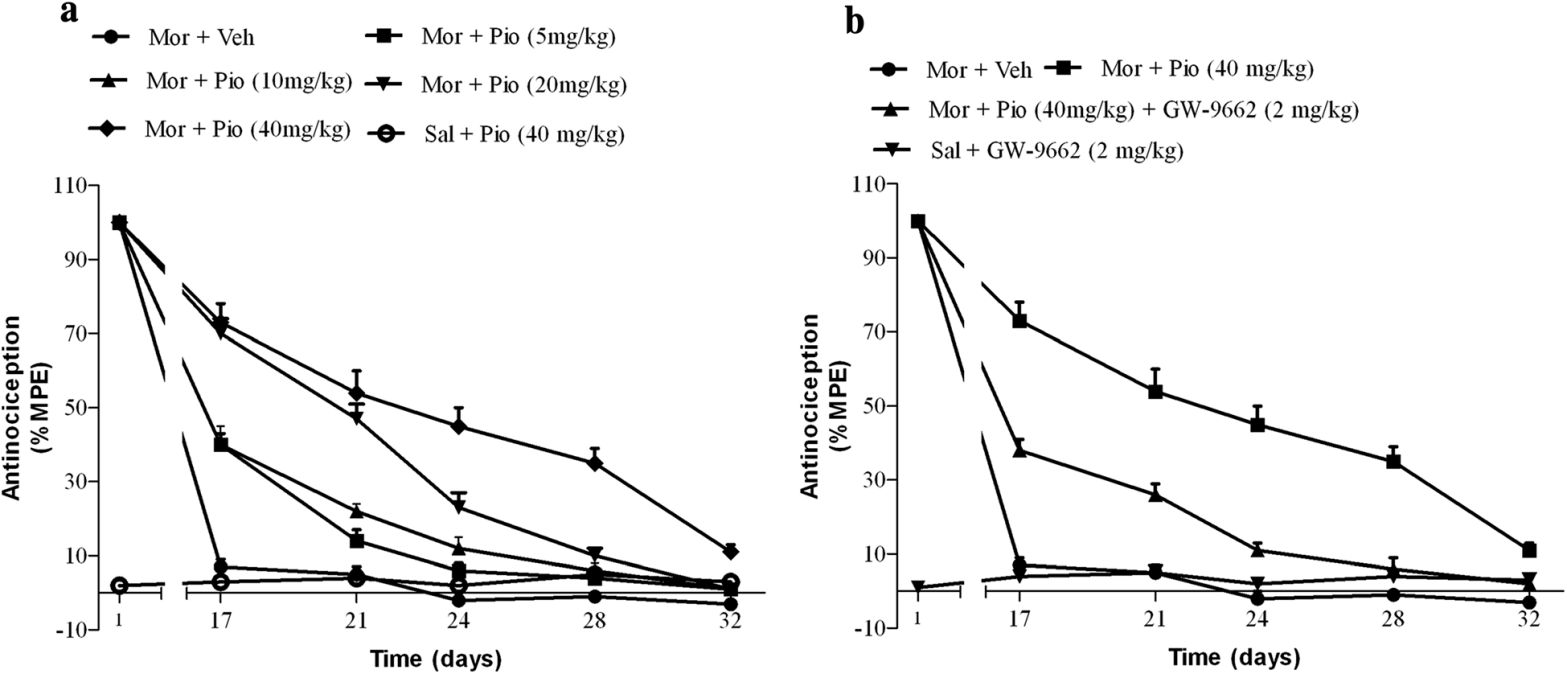
The effect of pioglitazone (5, 10, 20, 40 mg/kg; **a**) and its combination with GW-9662 (2 mg/kg; **b**) on the completion of morphine analgesic tolerance [2]

One part of our recently published finding indicated that pioglitazone attenuated the development of tolerance, shifting the first day of established tolerance from the 17^th^ day in the control group to the 32^th^ day (Fig. 1a, b). On the other hand administration of the GW-9662, the selective PPAR- γ antagonist, reversed the above mentioned effect of the pioglitazone [2].

Considering the central role of the inflammation in the morphine-induced tolerance and the anti-inflammatory feature of the pioglitazone, we considered to investigate the role of the pioglitazone in the morphine pharmacology focusing on the relation of the spinal pro-inflammatory cytokines changes and the attenuating effect of the pioglitazone on the morphine-induced tolerance.

## Methods

### Animals

Male Wistar rats (200–250 g) were received from the Pasteur Institute (Tehran, Iran). The animals were maintained under standard temperature (22 ± 0.5 °C) and light in conditions (12-h light/12-h darkness) with free access to food and water for 2 days before experiment. All experiments were executed in accordance with the Guide for Care and Use of Laboratory Animals of Tabriz University of Medical Sciences, Tabriz, Iran (National Institutes of Health Publication No 85–23, revised 1985).

### Drugs

Morphine sulfate (Daroupakhsh Company, Tehran, Iran) was dissolved in the normal saline and injected intraperitoneally (i.p). The pioglitazone hydrochloride (Zahravi Company, Tabriz, Iran) was suspended in the propylene glycol and distilled water (60:40) with a few drops of dimethylsulfoxide and administered orally 30 min before morphine injection. The PPAR-γ antagonist, GW-9662 (Sigma Chemical Company, USA), was dissolved in the pioglitazone vehicle and administrated subcutaneously (s.c). In this regard, the GW-9662 was administrated 30 min before the pioglitazone oral administration. Pioglitazone, morphine and GW-9662 doses were chosen based on our previous studies [1, 2].

### Experimental groups

Rats were divided into four experimental groups (*n* = 6–8) randomly. The experimental groups consisted of one saline treated group and three morphine treated groups. The saline treated group (vehicle) was only treated with saline and the vehicle. Thirty minutes before morphine injection, rats in the control group were received vehicle (0.5 mL) and the pioglitazone group were received pioglitazone (40 mg/kg) orally. To evaluate the role of PPAR-γ in pioglitazone effects on the morphine antinociception tolerance and the spinal pro-inflammatory cytokines, the GW-9662 (2 mg/kg, s.c) was administered 30 min before pioglitazone administration in the GW-treated group.

### Induction of tolerance to the analgesic effect of morphine

In order to induce tolerance to the analgesic effect of the morphine, the rats were received morphine (10 mg/kg, i.p) once a day until the tolerance completion on the control group [2]. It is worth noting that the experiment was carried out until day 18 of the experiment (one day after tolerance completion of the control group).

### Assessment of nociception

A radiant heat tail flick apparatus (model 37360; Ugo Basile, Italy) was used for assessment of the nociception. To this aim, the animals were gently restrained by hands during the test and the light beam (power intensity =5) was focused on 3–4 cm from the tail distal end. The tail flick latency was considered as the time between the tail exposure to the radiant heat and the tail withdrawal. The baseline tail flick latency was determined for each rat and designed as the baseline latency. The baseline latency was the average of three measurements and the intensity of the light was adjusted so that the baseline latencies were 2–3 s. A cut-off time (15 s) was imposed to prevent the tissue damage. The tail flick response latencies were defined as the percentage of the maximal possible effect (MPE%) using the following equation:

$$ MPE\%=\frac{Post\; drug\; latency\kern0.5em -\kern0.5em  Baseline\kern0.5em  latency}{Cutoff\kern0.5em  time\kern0.5em -\kern0.5em  Baseline\kern0.5em  latency}\times \kern0.5em 100 $$

The baseline latency was determined daily before each drug or vehicle administration. Moreover, vehicle or pioglitazone were gavage to the rats. It is worth noting that, thirty minutes before the pioglitazone administration, the rats were received the GW-9662. Thirty minutes later, the morphine (10 mg/kg) or the saline were injected intraperitoneally and finally the post-drug latency was measured after 30 min.

### Tissue preparation

On the eighteenth day (one day after the tolerance completion of the control group), two hours after the last dose of the morphine administration, the animals were rapidly (<1 min) sacrificed. Consequently, the lumbar spinal cord segments were removed, weighed, frozen in the liquid nitrogen and stored at -80 °C until being used for cytokine and NF-κB p 65 measurements. The frozen tissue samples were homogenized in ice-cold lysis buffer (500 mM Tris–HCl, pH 7.4, 150 mM NaCl, EDTA 0.5 mM, n-octyl-ß-D-glucopyranoside 1.5 % w/v) containing a complete protease inhibitor cocktail (Roche Cat # 11873 580 001) and were centrifuged at 14,000 rpm for 10 min at 4 °C [22]. The supernatants were used for analysis of TNF-α, IL-1ß and IL-6.

### Measurement of the spinal pro-inflammatory cytokine level

The spinal level of cytokines were determined using commercial ELISA kit specified for the rat IL-6 and TNF-α (eBioscience, Australia) and IL-1β (Glory Science Co., Ltd, USA) according to the manufacturer instruction. The results were expressed as picogram (p) per gram tissue.

### Determination of the spinal NF-κ B p65 activity

Briefly, the weighted lumbar spinal tissue (40 mg) was cut into small pieces and in the presence of phosphatase inhibitors (Cayman, item number # 10009305), detergent (Nonidet P-40 10 %; Cayman, item number # 600009) and the hypotonic buffer (Cayman, item number # 10009301), tissue was homogenized using pre-chilled Dounced homogenizer. Hypotonic buffer causes the cells to swell with breakage in the cell membrane by detergent, allowing access to the cytoplasm fraction while maintaining the integrity of the nuclear membrane. Subsequently, the nuclei fraction was separated from the cytoplasmic fraction by centrifugation. Then nuclear fraction was resuspended in ice-cold nuclear extraction buffer (Cayman, item number # 10009304) containing protease (Cayman, item number # 10009303) and phosphatase inhibitors. The suspension was vortexed; followed by gentle rocking on ice for 15 min (This step was repeated for five times). Then, the suspension was centrifuged at the 14000 rpm for 10 min at 4 °C. The supernatant was used for determining total protein concentration and NF-κB p 65 activity. A specific double stranded DNA sequence containing the NF-κB response element was immobilized onto the bottom of 96 well plates (Cayman, item number # 10007889). NF-κB contained in a nuclear extract, binds specifically to the NF-κB response element. NF-κB (p65) was detected by addition of specific primary antibody directed against NF-κB (p65). A secondary antibody conjugated to HRP was added to provide a sensitive colorimetric readout at 450 nm.

### Statistical analysis

Data were analyzed by the Statistical Product for Social Sciences (SPSS version 11.5). All data obtained are presented as the mean of six to eight rats ± SEM (standard error of the mean). For the behavioural data and the spinal NF-κB p65 activity, the difference between groups was determined by the one-way analysis of variance (ANOVA) followed by the Newman-Keuls test. The spinal cytokines levels, obtained from ELISA, were analyzed, using the one-way ANOVA followed by the Tukey’s post hoc test. The differences amongst means were considered statistically significant if *p* value < 0.05.

## Results and discussion

### Effect of pioglitazone on the development of morphine-induced tolerance

**Fig. 2:**
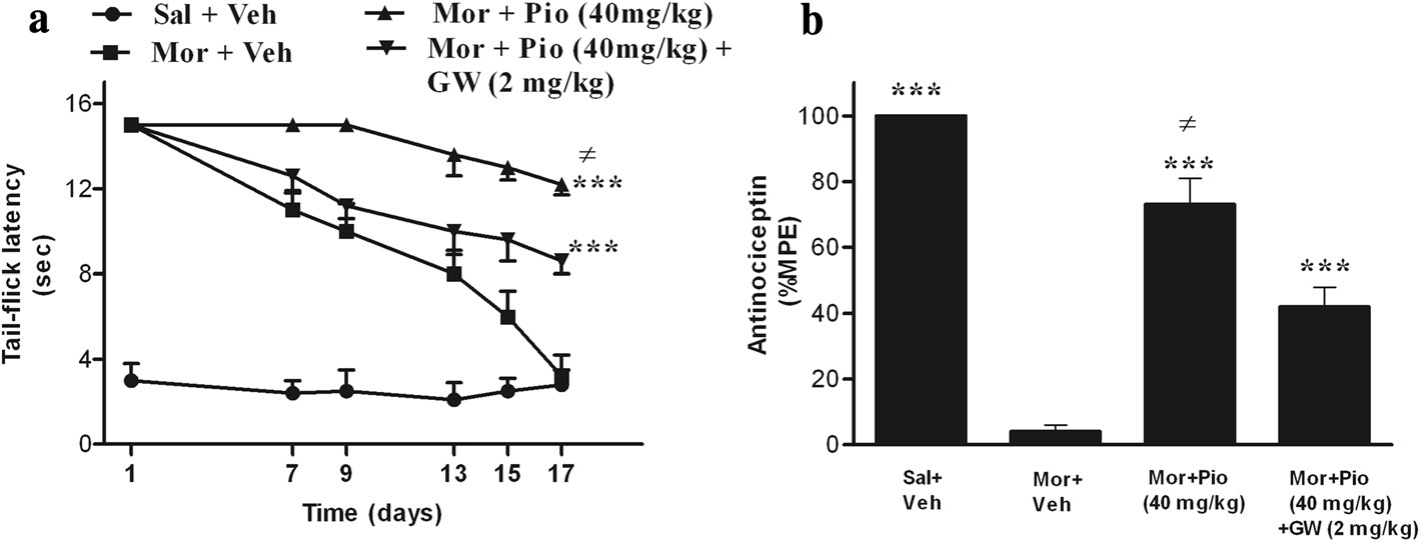
The tail-flick latency was measured every day 30 min after *i.p* injection of saline or morphine (**a**). The antinociceptive effect of 10 mg/kg (*i.p*) morphine on the day 18 in the groups that received saline, vehicle, pioglitazone (40 mg/kg), or GW-9662 (2 mg/kg) for 17 days (**b**). All date points are expressed as the mean ± SEM. ^***^: *p* < 0.001 compared with morphine-vehicle treated animals. ^≠^: *p* < 0.05 compared with GW-9662 (2 mg/kg) treated animals. (Sal: Saline; Mor: morphine; Pio: pioglitazone; GW: GW-9662; Veh: vehicle; MPE%: maximal possible effect)

The maximum analgesic effect of the morphine was recorded on the first day of the experiment. After that the morphine tail-flick latency threshold was progressively decreased to the base line levels in the control group and developed the tolerance on day 17 (Fig. 2a). Co-administration of the pioglitazone (40 mg/kg) with the morphine could hamper the morphine analgesic tolerance development significantly (*p* < 0.001; Fig. 2a). Moreover, as depicted in Fig. 2a administration of the GW-9662 (2 mg/kg) 30 min before administration of the pioglitazone, reversed the anti-tolerance effect of the pioglitazone (*p* < 0.05), significantly. On the day 18 of the experiments, the %MPE of the morphine in the rats that treated with the pioglitazone was 73 % which was significantly (*p* < 0.001) more that the %MPE of the control group (Fig. 2b). However, daily co-administration of PPAR-γ antagonist with the pioglitazone prevented the pioglitazone effect on the %MPE of morphine significantly (*p* < 0.05). These findings were consistent with our previous study [2] in that the oral administration of the pioglitazone (5, 10, 20 and 40 mg/kg) dose-dependently prevented the morphine antinociception tolerance completion (Fig. 1a) and the GW-9662 antagonized the anti-tolerance effect of the pioglitazone in the rat (Fig. 1b).

### Cytokines levels

**Fig. 3:**
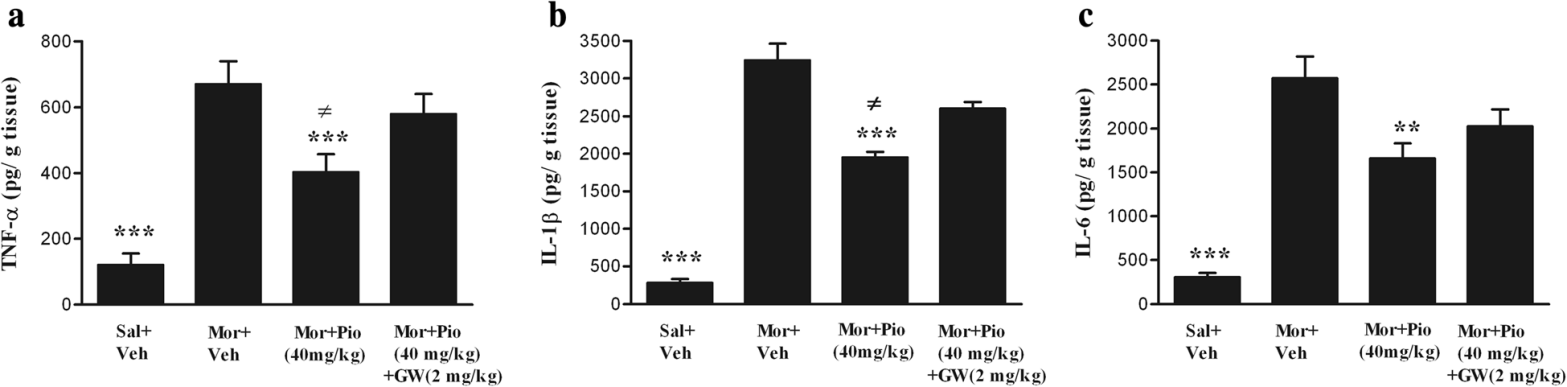
The levels of spinal pro-inflammatory cytokines in the different groups. Cytokines (**a**: TNF-α, **b**: IL-1β, **c**: IL-6) following 17 days treatment with morphine (10 mg/kg). All date points are expressed as the mean ± SEM. (Sal: Saline; Mor: morphine; Pio: pioglitazone; GW: GW-9662; Veh: vehicle).

The spinal levels of TNF-α, IL-1β and IL-6 increased significantly in the control group compared with the saline-vehicle treated group (*p* < 0.001 for all). As shown in the Fig. 3, concurrently oral administration of the pioglitazone (40 mg/kg) significantly attenuated the increases in the spinal TNF-α (*p* < 0.001), IL-1β (*p* < 0.001) and IL-6 (*p* < 0.01) protein levels compared with the control group. Moreover, administration of the GW-9662 (2 mg/kg) thirty minutes prior to the pioglitazone (40 mg/kg) reversed the pioglitazone effect on the spinal TNF-α (*p* < 0.05), IL-1β (*p* < 0.05) and IL-6 (*p* > 0.05) protein levels.

### Effect of pioglitazone or GW-9662 on the spinal NF-κB p65 activity

**Fig. 4:**
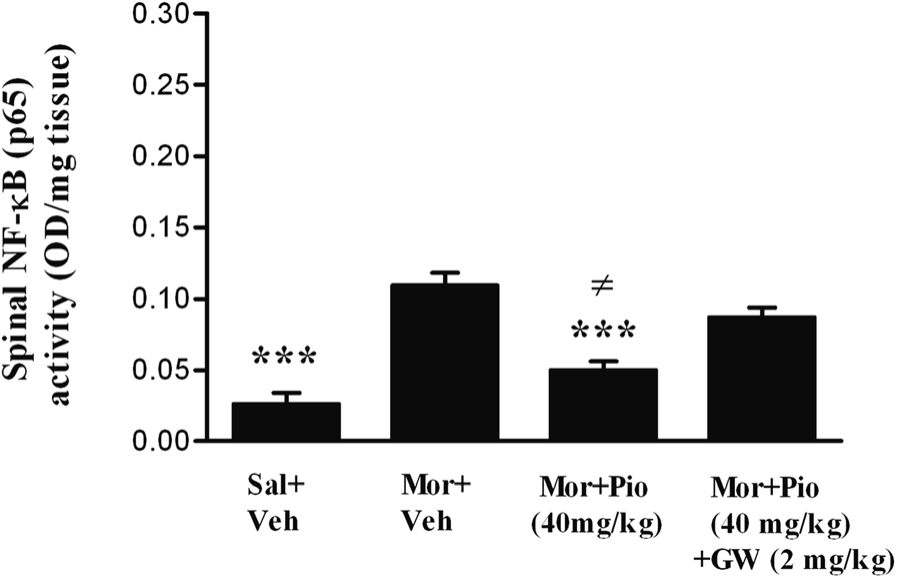
The activity of NF-κB p65 in different experimental group on day 18. All data points are expressed as the mean ± SEM. (Sal: Saline; Mor: morphine; Pio: pioglitazone; GW: GW-9662; Veh: vehicle)

As compared to the saline treated animals, morphine increased (319 %) the spinal NF-κB p65 activity (*p* < 0.001), significantly. As depicted in the Fig. 4, co-administration of the morphine with the pioglitazone (40 mg/kg) attenuated the spinal NF-κB p65 activity significantly compared with the control group (*p* < 0.001). Subcutaneous injection of the selective PPAR-γ antagonist, before the pioglitazone administration, prevented the pioglitazone inhibitory effect on the NF-κB p65 activity and enhanced its activity by 74 % (*p* < 0.01) versus the pioglitazone (40 mg/kg) plus morphine treated group.

In the present study we aimed to investigate the effect of the pioglitazone as a PPAR-γ agonist on the inflammatory responses developed through tolerance to morphine analgesia. Our results suggested that the pioglitazone (40 mg/kg) administration in morphine multiple-injected rats, attenuated the development of the morphine analgesic tolerance and we suggest that the improvement in the morphine effectiveness seen by the pioglitazone is related to the pioglitazone action on the PPAR-γ resulting in reduction of the pro-inflammatory responses. Moreover, our study indicated that the spinal pro-inflammatory cytokines play an important role in the development of morphine antinociception tolerance. Besides, our recently finding indicated that morphine augments both pro-inflammatory cytokines (e.g., TNF-α, IL-1β and IL-6) and NF-κB activity in the cerebral cortex of the rat, which was consistent with the behavioural manifestation of morphine analgesic tolerance. However, concurrent administration of the pioglitazone (40 mg/kg) hampered these effects of the morphine [23].

Morphine-induced tolerance is associated with a significant increase in the glial derived pro-inflammatory cytokines in the rat spinal cord. It has been established that targeting these cytokines [12], attenuating the glial activity [11], antagonizing the spinal IL-1β receptors [5] or interrupting the NF-κB activity [13] lead to improving the morphine effectiveness and delaying the onset of analgesic tolerance. It is also indicated that the PPAR-γ agonists exert anti-inflammatory effects through the suppression of the glial cell function and the regulation of the activity of different transcription factors such as the NF-κB [15, 17, 24]. Our recently study indicated that pioglitazone attenuates the development of tolerance in the rat [2]. Moreover, acute administration of pioglitazone attenuated the naloxone induced morphine withdrawal syndrome in rat [1].

This is highly confirmed that in inflammatory processes, the NF-κB is the most important transcription factor, which controls the promoter regions of the different pro-inflammatory cytokine genes those are implicated in the morphine antinociception tolerance. The NF-κB p65 activity at the lumbar spinal cord in the pioglitazone-treated rats have been measured and compared with equivalent measurements in the morphine-injected animals. We assessed whether the NF-κB might play a functional role in the impact of the pioglitazone on the improvement of the morphine effectiveness. Herein, we report that oral administration of the pioglitazone reduced the NF-κB p65 activity in the lumbar spinal cord. Consistent with these results, Wang et al. (2010) reported that interruption in NF-κB transcription activity ameliorated the morphine analgesic tolerance development [13]. Furthermore, the GW-9662 as the PPAR-γ antagonist reversed the inhibitory effects of the pioglitazone on the NF-κB p65 activity. This finding was in consistent with the recent findings that the pioglitazone inhibited the NF-κB activity and the GW-9662 reversed its effects [19]. One of the numerous mechanisms that support the pro-inflammatory cytokines involvement in the morphine analgesic tolerance, is association of increased glial derived pro-inflammatory cytokines levels in the rat spinal cord to tolerance [5]. The glial derived pro-inflammatory cytokines lead to the morphine analgesic tolerance either directly or indirectly. Directly these inflammatory substances initiate the downstream signaling transduction cascade and aggravate the release of prostaglandins, nitric oxide and glutamate therefore, promote pain transmission [25]. Indirectly, the glial derived pro-inflammatory cytokines down regulate the glutamate transporters and produce a high glutamate amount in synapses [4, 10]. Earlier studies have reported that the TNF-α leads to an immense activation of the glutamatergic system in the rat spinal cord through down-regulation of the glutamate transporters and exocytosis of the NMDA receptors [10]. Hyperactivation of the glutamatergic system resulted in enhancing the intracellular mechanisms of morphine tolerance [26]. It is worth noting that activation of the NMDA receptors is associated with the morphine analgesic tolerance [3]. We found that the pioglitazone has a remarkable ability in inhibiting the production of pro-inflammatory cytokines including TNF-α, IL-1β and IL-6 where the GW-9662 reverses its effect.

In addition to the anti-inflammatory properties of the pioglitazone, it is found that the pioglitazone is capable of reducing the NMDA receptors mediated calcium current and potential conferring the neuroprotection [27]. In this regard, it is widely indicated that the NMDA receptor antagonist can inhibit the morphine tolerance.

## Conclusions

Administration of the pioglitazone with the morphine attenuated both development of the morphine tolerance and the spinal pro-inflammatory cytokines expression. However, these effects of the pioglitazone were abolished by the PPAR-γ selective antagonist (GW-9662). The effects of the pioglitazone might be caused, at least in part, through the PPAR-γ on suppression of the NF-κB activity in the spinal cord, which resulted in negative regulation of the IL-6, IL-1β and TNF-α expression.
